# *Pseudomonas* species prevalence, protein analysis, and antibiotic resistance: an evolving public health challenge

**DOI:** 10.1186/s13568-022-01390-1

**Published:** 2022-05-09

**Authors:** Ayman Elbehiry, Eman Marzouk, Musaad Aldubaib, Ihab Moussa, Adil Abalkhail, Mai Ibrahem, Mohamed Hamada, Wael Sindi, Feras Alzaben, Abdulaziz Mohammad Almuzaini, Abdelazeem M. Algammal, Mohammed Rawway

**Affiliations:** 1 Department of Public Health, College of Public Health and Health Informatics, Qassim University, Al-Bukairiyah, Saudi Arabia; 2 Department of Bacteriology, Mycology and Immunology, Faculty of Veterinary Medicine, University of Sadat City, Sadat City, Egypt; 3 Department of Veterinary Medicine, College of Agriculture and Veterinary Medicine, Qassim University, Buraydah, Saudi Arabia; 4 Department of Botany and Microbiology, College of Science, King Saud University, P.O. Box 2455, Riyadh, 11451 Saudi Arabia; 5 Department of Microbiology, Faculty of Veterinary Medicine, Cairo University, Giza, Egypt; 6 Department of Public Health, College of Applied Medical Science, King Khalid University, Abha, Saudi Arabia; 7 Department of Fish Diseases and Management, Faculty of Veterinary Medicine, Cairo University, Cairo, Egypt; 8 Department of Food Hygiene & Control, Faculty of Veterinary Medicine, Sadat City University, Sadat City, Egypt; 9 Department of Preventive Medicine, King Fahad Armed Forces Hospital, Jeddah City, Saudi Arabia; 10 Department of Bacteriology, Immunology, and Mycology, Faculty of Veterinary Medicine, Suez Canal University, Ismailia, 41522 Egypt; 11 Biology Department, College of Science, Jouf University, Sakaka, Saudi Arabia; 12 Botany and Microbiology Department, Faculty of Science, Al-Azhar University, Assiut, Egypt

**Keywords:** *Pseudomonas* spp., Chilled meat, Identification, Antimicrobial resistance

## Abstract

Psychrotrophic *Pseudomonas* is one of the significant microbes that lead to putrefaction in chilled meat. One of the biggest problems in the detection of *Pseudomonas* is that several species are seemingly identical. Currently, antibiotic resistance is one of the most significant challenges facing the world's health and food security. Therefore, this study was designed to apply an accurate technique for eliminating the identification discrepancy of *Pseudomonas* species and to study their resistance against various antimicrobials. A total of 320 chicken meat specimens were cultivated, and the isolated bacteria’ were phenotypically recognized. Protein analysis was carried out for cultured isolates via Microflex LT. The resistance of *Pseudomonas* isolates was recorded through Vitek® 2 AST-GN83 cards. Overall, 69 samples were identified as *Pseudomonas* spp. and included 18 *Pseudomonas lundensis* (*P. lundensis*), 16 *Pseudomonas fragi* (P. fragi), 13 *Pseudomonas oryzihabitans* (*P. oryzihabitans*), 10 *Pseudomonas stutzeri* (*P. stutzeri*), 5 *Pseudomonas fluorescens* (*P. fluorescens*), 4 *Pseudomonas putida* (*P. putida*), and 3 *Pseudomonas aeruginosa* (*P. aeruginosa*) isolates. Microflex LT identified all *Pseudomonas* isolates (100%) correctly with a score value ≥ 2.00. PCA positively discriminated the identified isolates into various groups. The antimicrobial resistance levels against *Pseudomonas* isolates were 81.16% for nitrofurantoin, 71% for ampicillin and ampicillin/sulbactam, 65.22% for cefuroxime and ceftriaxone, 55% for aztreonam, and 49.28% for ciprofloxacin. The susceptibilities were 100% for cefotaxime, 98.55% for ceftazidime, 94.20% for each piperacillin/tazobactam and cefepime, 91.3% for cefazolin. In conclusion, chicken meat was found to be contaminated with different *Pseudomonas* spp., with high incidence rates of *P. lundensis*. Microflex LT is a potent tool for distinguishing *Pseudomonads* at the species level.

## Introduction

The nutrients in meat, including proteins, essential fatty acids, vitamins, and minerals, make it one of the most important food sources. As well as being highly active in water, it is highly susceptible to spoilage by microorganisms (Wickramasinghe et al. 2019). During and after the slaughtering process, meat can become contaminated with microbes (Bantawa et al. 2018). As strict procedures exist to ensure quality in the meat industry, the transportation and storage of meat has become a major source of contamination with microbes that harm human health (Kennedy et al. 2005; Nychas et al. 2008; Rouger et al. 2017). Meat and its products carry various microbiological dangers, which significantly increase the risk of infection and death, especially in developing countries (Lianou et al. 2017).

Spoilage bacteria are commonly found in raw meat before the slaughter process, and they can invade the meat during the handling, carriage, or packing process. (Doulgeraki et al. 2012). Food processing can be a main source of the food chain contamination, particularly for fresh foods or products that are not subjected to heat treatments or other sanitization during its preparation. *Pseudomonas* is an adaptable microorganism in the food processing environment (Stellato et al. 2017). As a source of meat contamination with *Pseudomonas,* poor sanitation of meat shops and unhygienic processing and unawareness of meat retailers of basic requirements and guidelines of meat shop, can be identified (Bantawa et al. 2018).

Among the important microbes that lead to the contamination of meat and meat products are *Pseudomonas*, *Brochothrix, Acinetobacter,* and *Shewanella*, which have been recovered from frozen meat (Wickramasinghe et al. 2019). Of the genus *Pseudomonas*, *P. fluorescens*, *P. fragi*, *P. lundensis*, *P. migulae*, and *P. putida* are considered frequent species found in chilled meat (Doulgeraki et al. 2012). In general, most of *Pseudomonas* species can grow at temperatures below 7 °C (psychrotrophic) and are characterized by their capabilities to contaminate fresh food and the surrounding environment, especially in the absence of the necessary sterilization procedures (Stellato et al. 2017; Quintieri et al. 2019). *Pseudomonas lundensis* (*P. lundensis*) represents one of the most significant psychrotrophic microorganisms that leads to deterioration in frozen meat (Liu et al. 2015). *P. lundensis* is a gram-negative psychrotrophic motile bacterium that can grow at temperatures fluctuating from 0 to 33 °C (Ercolini et al. 2010). In addition, previous studies carried out by Caldera et al. (2016) and Raposo et al. (2017) demonstrated that most *Pseudomonas* species have the capacity to release different thermotolerant proteolytic and lipolytic enzymes that can seriously decrease the quality and shelf life of meat and its products.

Although its ability to produce fluorescent pigments, *P. lundensis* may appear to be confusing when compared to non-pigment species (such as *P. fragi*). Further studies have revealed *P. lundensis* to be strictly associated with both the *P. fragi* and the *P. fluorescens* groups. During the stowage process, these bacteria form unpleasant odours and slime, causing meat to deteriorate (Wickramasinghe et al. 2019). Nevertheless, *P. lundensis* has predominantly been associated with milk and meat putrefaction, and new studies have utilized culture techniques and established that it exists in patients’ lungs, particularly in patients suffering from cystic fibrosis (Scales et al. 2018). Nonetheless, the role of *P. lundensis* in lung deterioration and its probable role in respiratory distress are still unidentified. It is worth noting that in contrast to other microbes that cannot grow at low temperatures, psychrotrophic Pseudomonads have the ability to adapt and rapidly colonize ice-cold foods, leading to deterioration and biofilm formation (De Jonghe et al. 2011; Quintieri et al. 2019; Orellana-Saez et al. 2019). This later action increases their adaptability and scattering capacity, and as a result, measures to eliminate them can provoke and increase their resistance to various antimicrobial drugs. As a consequence of these features, the existence of *Pseudomonas* spp. such as *P. lundensis*, *P. fragi*, *P. fluorescens*, *P. gessardii*, and *P. taetrolens* in hilled fresh food products has been developing great attention (Baruzzi et al. 2012; Guidone et al. 2016; Brasca et al. 2018; Quintieri et al. 2019).

Nevertheless, culture-independent techniques supply a broader viewpoint on bacterial assortment, and culturing novel isolates remains significant and habitually accomplished in the majority of microbiological laboratories. This is predominantly correct for ecological bacteria, which are considered a huge source of new natural products for feed flavours, and in the field of developing medicines and other manufacturing products (Stafsnes et al. 2013; Timperio et al. 2017). To recognize and categorize new isolates at the genus and species levels, numerous techniques, such as phenotypic and genotypic methods, exist for this purpose. However, while these methods are considered the gold standard for the identification of different types of microorganisms, they cannot provide adequate and reliable data regarding the detection and discrepancy of various bacteria at the species level (Pesciaroli et al. 2015).

Therefore, it is necessary to use a fast and accurate technique such as mass spectrometry technology to recognize and distinguish different microbes isolated from food products. MALDI-TOF MS is an extraordinary throughput-dependent tool utilized for protein analysis. Previous studies have proven that the analysis of complete bacterial cells using the technique of protein analysis is one of the most important methods employed in identifying different microbes in the past ten years. (Elbehiry et al. 2019). However, the challenge in the application of this technology in bacterial identification and grouping is the accessibility of cost-effective devices delivered with powerful datasets and easy software (Emonet et al. 2010; Elbehiry et al. 2017).

Nevertheless, the initial utilization of MALDI-TOF MS for the detection of several microorganisms, including bacteria and fungi, was not commonly used 30 years ago due to the absence of satisfactory databases (Tshikhudo et al. 2013). Recently, MALDI-TOF MS has been able to classify and discriminate psychrotrophic bacteria at both the genus and species levels by matching the spectral profiles of field isolates with stored spectral proteins from reference strains (Vithanage et al. 2014; Dong 2020). The workflow of MALDI-TOF MS is based mainly on dissolving a fresh bacterial colony in an appropriate matrix compound and then inoculating it onto a target plate for protein analysis using laser shots in a measuring chamber. As a final point, a mass spectrum is obtained and displayed using particular software (Elbehiry et al. 2019).

Recently, antimicrobial resistance of different microorganisms has become an urgent matter due to their direct impact on public health worldwide. It is known that antibiotic-resistant bacteria have a close relationship with infections in hospitals, and *P. aeruginosa* is one of the most important opportunistic bacteria that affect human health, particularly in patients with defective immune systems (Chatterjee et al. 2016; Quintieri et al. 2019).

Furthermore, current suggestions emphasize that nonvirulent *Pseudomonads* not only have the ability to cause infections in the bloodstream of humans but also exhibit numerous types of multidrug resistance against various classes of antibiotics, which is a source of great danger to human health as a result of their high adaptability (Chatterjee et al. 2016; Cole and Singh 2017). Numerous *Pseudomonas* spp. of food origin are competent to resist various antimicrobial agents of various classes, especially β-lactams such as penicillins, cephalosporins, carbapenems, and monobactams (King et al. 2014). The objective of the present investigation was to identify *Pseudomonas* spp. recovered from chicken meat samples using a Microflex LT device and to examine the antimicrobial resistance of *Pseudomonas* against various antimicrobial agents using AST GN83 cards.

## Materials and methods

### Sample collection

A total of 320 frozen chicken meat products (200 g of each sample) represented by breast, thigh, burger, and nuggets (80 of each) were randomly collected from various retail stores in the Al-Qassim region, Saudi Arabia, at various intervals from June to December 2020. Each sample was preserved independently in a Ziploc® brand freezer bag at 4 ºC, and all samples were then transferred directly to the microbiology laboratory in a heat-insulated ice box under the appropriate hygienic conditions for bacteriological examination.

### Sample processing and isolation of Pseudomonas spp.

According to the guidelines provided by the Feng et al. (2002), 25 g of each sample was moved into a sterilized container with 225 ml of sterile peptone water (0.1%) under strict hygienic measures, and homogenization was performed using a Denville Ultra EZgrind™ Tissue Homogenizer (Thomas Scientific, USA) at 14,000 rpm for 3 successive minutes and was then incubated for 5 min at 25 °C. From this mixture, 1 ml was moved into a sterilized test tube containing 9 ml of sterile peptone water, from which tenfold (1:10) serial dilutions were processed. The prepared samples were subjected to isolation and determination of *Pseudomonas* counts (ISO 2003). In brief, 0.1 ml of each homogenized sample was independently inoculated into duplicate Petri dishes on Thermo Scientific™ Remel™ *Pseudomonas* Isolation Agar supplemented with glycerol and uniformly distributed with a sterile plastic spreader (Thomas Scientific, USA). The inoculated plates were incubated at 4 °C and 25 °C for a couple of days. Then, greenish yellow colonies were detected and enumerated. The average count was considered and recorded from serial dilutions of 10^–3^ to 10^–6^. Subculturing of all suspected colonies was carried out by streaking on nutrient agar and incubating at 4 °C for 3 to 5 days to obtain purified colonies. All purified strains were kept in the CRYOBANK™ Bacterial Culture Freezing System (COPAN Diagnostics Inc., Murrieta, USA) for further investigations.

### Phenotypic identification of Pseudomonas species

#### Microscopy and Gram stain properties

All purified strains kept in Cryobank vials were subcultured again for microscopic identification of *Pseudomonas* species using the Gram staining technique (Becerra et al. 2016).

In brief, a fresh colony of each strain was fixed onto a clean, dried slide, and then the colony was flooded with different chemicals. Crystal violet dye was first dropped onto the glass slide containing bacterial cells, and then iodine was added to fix the dye. Ethanol was then added to remove the dye from unspotted cells, and safranin was added as a final point to stain the gram-negative bacteria. The stained slides were scanned by light microscopy using an oil immersion lens to observe the morphology of bacteria. The isolates were recognized as gram-negative if they appeared pink.

### Enzymatic activities

The enzymatic activities of *Pseudomonas* species were evaluated using certain biochemical tests, including oxidase, citrate utilization and indole tests (LaBauve and Wargo 2012). The oxidase test was performed by smearing a fresh colony of each isolate onto a sterile oxidase filter paper disc (Sigma–Aldrich, USA) soaked in distilled water. The positive oxidase activity results are indicated by the appearance of a purple colour. The capacity of *Pseudomonas* spp. to utilize citrate as a source of energy was carried out by streaking a fresh purified colony onto a Simmons citrate agar (Sigma–Aldrich, USA) slant and incubated at 37 °C for 5–7 successive days. Citrate utilization was indicated by the appearance of a sky-blue colour. The capability of *Pseudomonas* spp. to convert tryptophan into indole was tested by inoculation of the suspected organism into tryptophan broth (Sigma–Aldrich Chemie GmbH, Germany), which was then incubated at 37 °C for a couple of days. Thereafter, using gentile agitation, 0.5 ml of Kovacs reagent was added until a red–violet colour appeared, indicating positive results, while a yellow colour indicates negative results.

### *Identification and determination of antimicrobial resistance *via* the Vitek 2 Compact system*

All gram-negative isolates that showed positive results for both citrate utilization and oxidase tests and negative indole tests were further examined by the Vitek 2 Compact system (bioMerieux, France) for confirmation of species identification and antimicrobial resistance. Briefly, preparation of the isolate suspension was carried out and then adjusted using McFarland standards (0.5 to 0.63) as stated in the company’s instructions. AST-GN83 gram-negative identification cards, which included 18 antimicrobial drugs, were utilized as gram-negative antibiotic susceptibility cards for *Pseudomonas* spp. (Table 1). According to the company’s instructions, Vitek®2 cards were inoculated, and then the isolate IDs were submitted to the device to permit the selection of precise interpretive standards. Based on the authorizations of the Clinical and Laboratory Standards Institute (CLSI) (Schreckenberger and Binnicker 2011), the minimum inhibitory concentration (MIC) was interpreted as susceptible, intermediate, or resistant. *Pseudomonas* sp. (ATCC® 19,151™) was utilized as a reference strain throughout the experiment. All strains that exhibited intermediate reactions against antimicrobial drugs were considered resistant strains.

**Table 1:** MIC ranges (µg/ml) of the antibiotics used by Vitek 2 Compact AST GN83 cards

Antimicrobial drug	MIC range (µg/ml)
Amikacin	2–64
Amoxicillin/Clavulanic Acid	2/1–32/16
Ampicillin	2–32
Ampicillin/Sulbactam	2/1–32/16
Aztreonam	1–64
Cefazolin	4–64
Cefepime	1–645
Cefotaxime	0.25–64
Cefoxitin	4–64
Ceftazidime	1–64
Ceftriaxone	0.25–64
Cefuroxime	1–64
Ciprofloxacin	0.25–4
Gentamicin	1–16
Meropenem	0.25–16
Nitrofurantoin	16–512
Piperacillin/Tazobactam	4/4–128/4
Trimethoprim/Sulfamethoxazole	20–320

### Proteomic screening for identification of Pseudomonas spp.

According to the method previously described by Barreiro et al. (2010), Microflex LT (Bruker Daltonics, Bremen, Germany) was applied for the recognition and classification of *Pseudomonas* species from chicken meat samples. All isolates were analysed using both FlexControl and Compass software (Flex Series version 1.3). All isolates were prepared by culturing on nutrient agar (Sigma–Aldrich, USA) followed by incubation for a couple of days at 37 °C. The ethanol/formic acid extraction procedure was utilized as stated by Bruker Daltonics. Briefly, 2 pure colonies were relocated onto a clean Eppendorf tube holding 300 µl of sanitized water and 900 µl of ethanol (99.9%). The contents were mixed carefully by centrifugation at 13,000 rpm for 2 mins. The obtained pellet was dried in air for 5 min after the removal of the supernatant. Fifty microlitres of formic acid (70%) was added to the air-dried pellet, and acetonitrile (70%) was then added after proper mixing for 2 min at 13,000 rpm. One microlitre of the supernatant for each isolate was then placed onto a target plate of Microflex LT and left to dry at 25 °C. Subsequently, 1 µl of matrix solution αcyano-4 hydroxy-cinnamic acid was added. The target plate was then submitted to the Microflex LT machine for identification and data reading. A bacterial test standard (*Escherichia coli*) was used as a positive control throughout the experiment.

The score values of indeterminate spectra were compared with the reference spectra stored in the databank. Microflex LT has the capacity to accurately recognize and distinguish different microorganisms when the values range from 2 to 3. However, misidentification can result if this value ≤ 1.69. The diverse spectra created by IVD Compass Software were analysed via a m/z range from 2000 Da to 20,000 Da. A dendrogram could be produced from the main spectral profile (MSP) database, which includes > 6.989 different species of bacteria and fungi.

## Results

### Frequency and counting of Pseudomonas spp.

Out of the 320 chicken meat samples involved in the current investigation, 69 (21.56%) were determined to be positive for *Pseudomonas* spp. using culturing techniques. Out of 69 positive isolates, 8 (11.59%), 11 (15.94%), 25 (36.23%), and 25 (36.23%) were isolated from breast, thigh, burger, and nugget meat, respectively. After statistical analysis, the mean values of colony-forming units (CFU/g) were 10.1 × 10^3^ ± 1.45 × 10^3^, 7.4 × 10^3^ ± 0.89 × 10^3^, 6.5 × 10^3^ ± 6.43 × 10^3^ and 4.3 × 10^3^ ± 7.56 × 10^3^ for the isolates recovered from the breast, thigh, burger, and nugget meat, respectively (Table 2).

**Table 2:** The mean values of colony forming units (CFU/g) for 69 *Pseudomonas* spp. detected in chicken meat products

Chicken meat product	Minimum	Maximum	Mean value ± Standard error
Breast (n = 8)	2.0 × 10^2^	10.1 × 10^3^	10.1 × 10^3^ ± 1.45 × 10^3^
Thigh (n = 11)	3.4 × 10^2^	7.4 × 10^3^	7.4 × 10^3^ ± 0.89 × 10^3^
Hamburger (n = 25)	3.9 × 10^2^	6.5 × 10^3^	6.5 × 10^3^ ± 6.43 × 10^3^
Nuggets (n = 25)	3.7 × 10^2^	4.3 × 10^3^	4.3 × 10^3^ ± 7.56 × 10^3^

### Biochemical analysis of Pseudomonas spp.

A total of 69 *Pseudomonas* isolates that showed positive results for oxidase and citrate utilization and negative results for indole were examined by the Vitek 2 Compact system. Consistent with the interpreted results, 18 (26.09%) strains were identified as *Pseudomonas lundensis* (*P. lundensis*), 16 (23.19%) *Pseudomonas fragi* (*P. fragi*), 13 (18.84%) *Pseudomonas oryzihabitans* (*P. oryzihabitans*), 10 (14.49%) *Pseudomonas stutzeri* (*P. stutzeri*), 5 (7.25%) *Pseudomonas fluorescens* (*P. fluorescens*), 4 (5.8%) *Pseudomonas putida* (*P. putida*), and 3 (4.35%) *Pseudomonas aeruginosa* (*P. aeruginosa*). From the previous results, it was indicated that *P. lundensis* was the most common *Pseudomonas* spp. recovered from chicken meat samples, followed by *Pseudomonas fragi, P. oryzihabitans,* and *P. stutzeri* (Table 3).

**Table 3:** *Pseudomonas* spp. recovered from chicken meat products

*Pseudomonas* spp.	Chicken meat products								Total	
	Breast		Thigh		Hamburger		Nuggets			
	No.	%	No.	%	No.	%	No.	%	No.	%
*P. lundensis*	3		2		7		6		18	26.09
*P. fragi*	1		3		7		5		16	23.19
*P. oryzihabitans*	3		2		5		3		13	18.84
*P. stutzeri*	0		3		3		4		10	14.49
*P. fluorescens*	1		0		2		2		5	7.25
*P. putida*	0		1		1		2		4	5.80
*P. aeruginosa*	0		0		0		3		3	4.35
*Total*	8		11		25		25			

### Proteomic analysis of Pseudomonas spp.

**Fig. 1:**
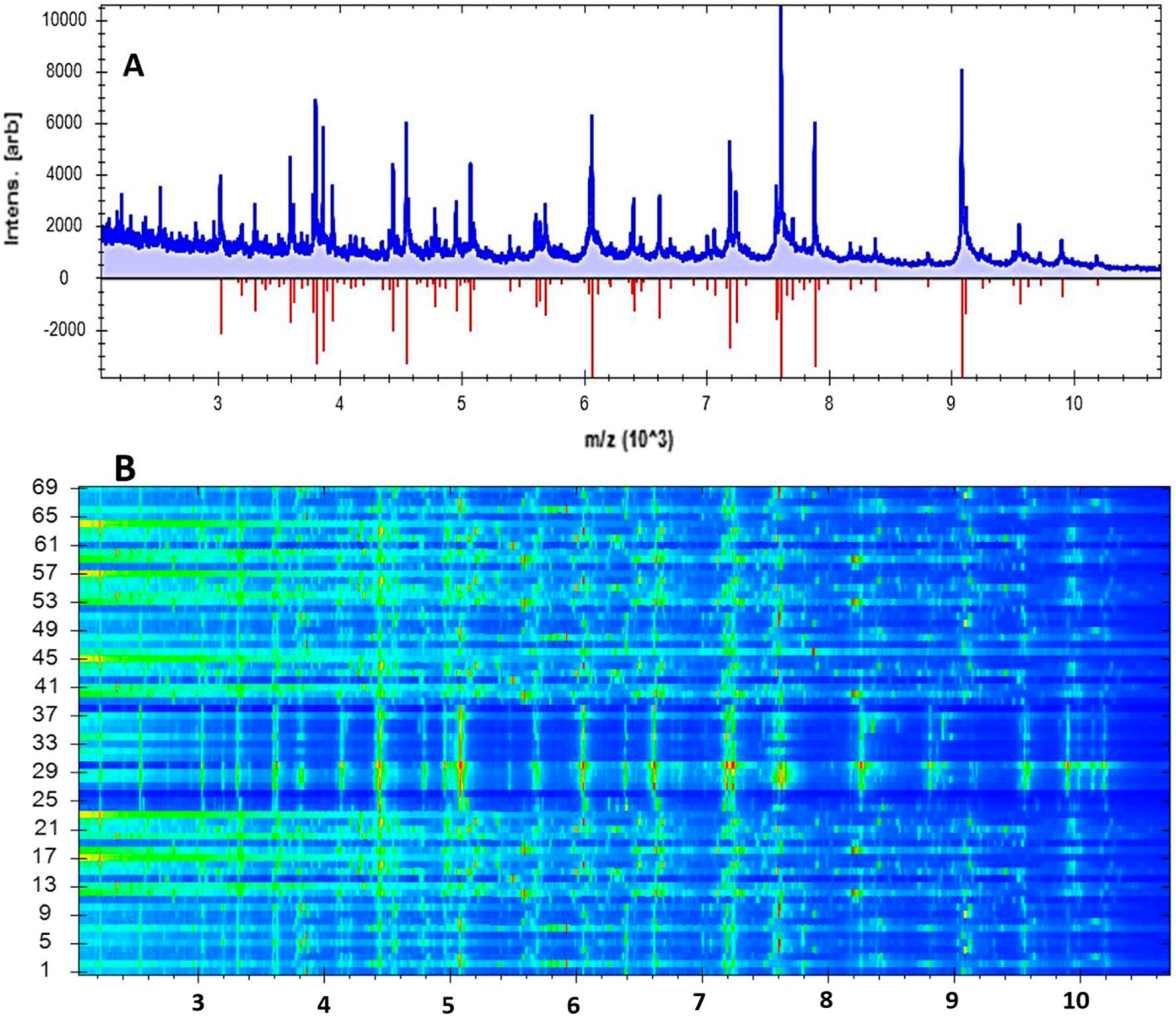
Scattering of spectral proteins of 69 *Pseudomonas* spp.; **a** Spreading of peak intensities with a mass charge ratio from 2000 to 10,200 Da; **b** A gel view, in which the wide-ranging colour of dots represents the assembly of different spectra

**Fig. 2:**
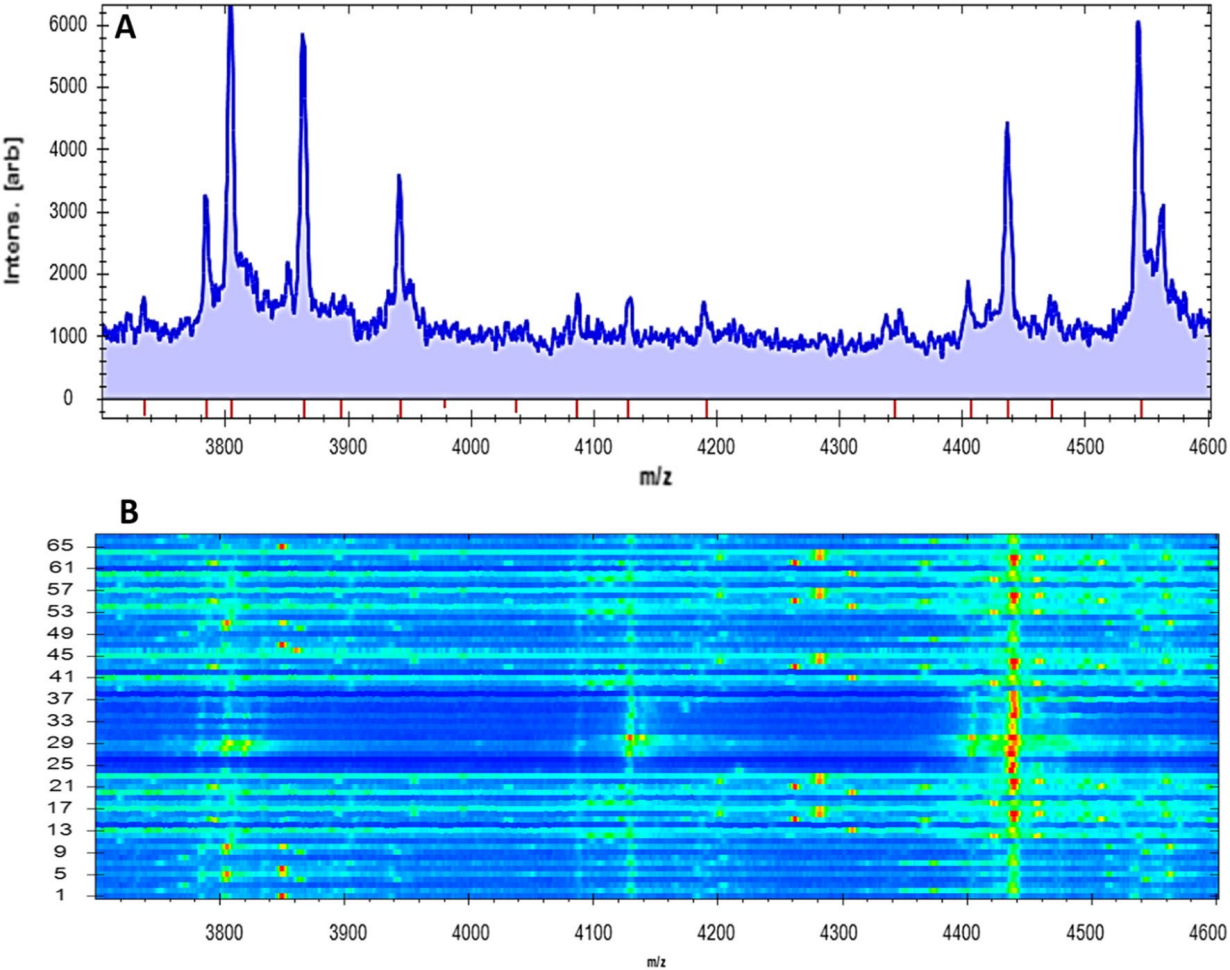
Scattering of spectral proteins of 69 *Pseudomonas* spp. **a** Higher peak intensities were distributed inside the line spectra with mass charge ratios from 3000 to 4600 Da; **b** A gel outline of spectral proteins scattered with the same pattern

**Fig. 3:**
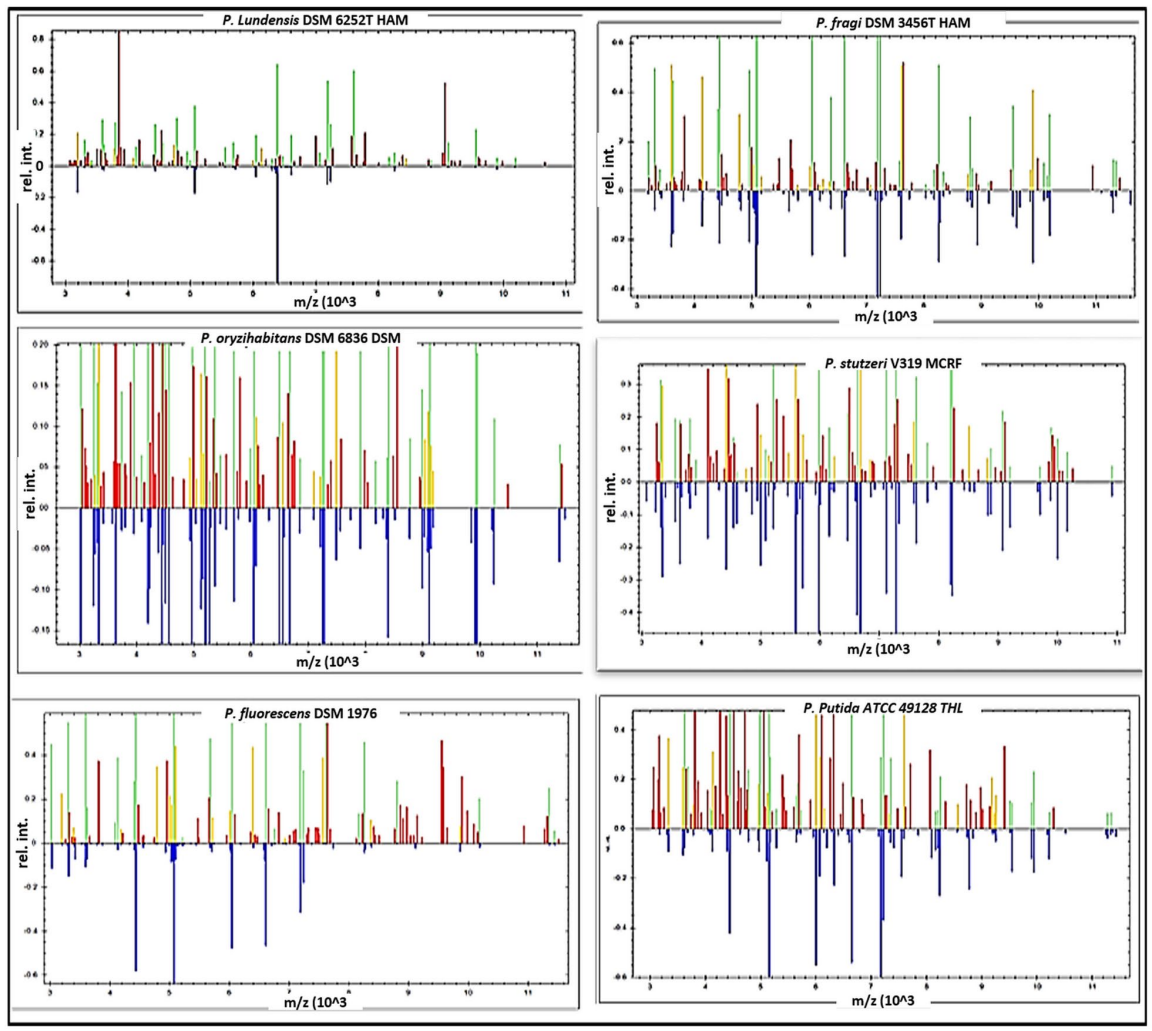
Mass spectral protein profiles of different *Pseudomonas* species recovered from chicken meat samples compared with 6 reference strains deposited in the IVD Compass software of Microflex LT. The stored spectral proteins are represented by blue colour in the lower part, while the green spectra in the upper part indicate matched peaks, red spectra indicate incompatible peaks, and yellow spectra indicate intermediate peaks

In the existing study, the isolated strains were screened by Microflex LT, and the spectra of the field isolates were parallel to the reference spectra. According to the results obtained, Microflex LT was capable of verifying all *Pseudomonas* spp. by 100%. Analysing these results shows that approximately 20 prominent ion peaks were detected in the original bands from the region varying from 2000 to 10,200 Daltons (Da) (Fig. 1A), which were confirmed from the gel view (Fig. 1B), and robust peaks were revealed at 3800, 3860, 4440, and 4550 Da (Fig. 2A), which were confirmed from the gel view (Fig. 2B). All 69 strains of *Pseudomonas* spp. were correctly identified as follows: 18 *P. lundensis*, 16 *P. fragi*, 13 *P. oryzihabitans*, 10 *P. stutzeri*, 5 *P. fluorescens*, 4 *P. putida*, and 3 *P. aeruginosa*. All of these species were synchronized with the *P. Lundensis* DSM 6252 T HAM (Fig. 3), *P. fragi* DSM 3456 T HAM, *P. oryzihabitans* DSM 6835 T, *P. stutzeri* V319 MCRF, *P. fluorescens* DSM 1976, *P. putida* ATCC 49,128 THL, and *P. aeruginosa* DSM 1117 reference strains stored in Compass IVD software. All *Pseudomonas* strains were distinguished by matching their spectra with the Bruker database, which includes 70 strains obtained from the American Type Culture Collection (ATCC) and the German Collection of Microorganisms and Cell Cultures GmbH (DSMZ).

**Fig. 4:**
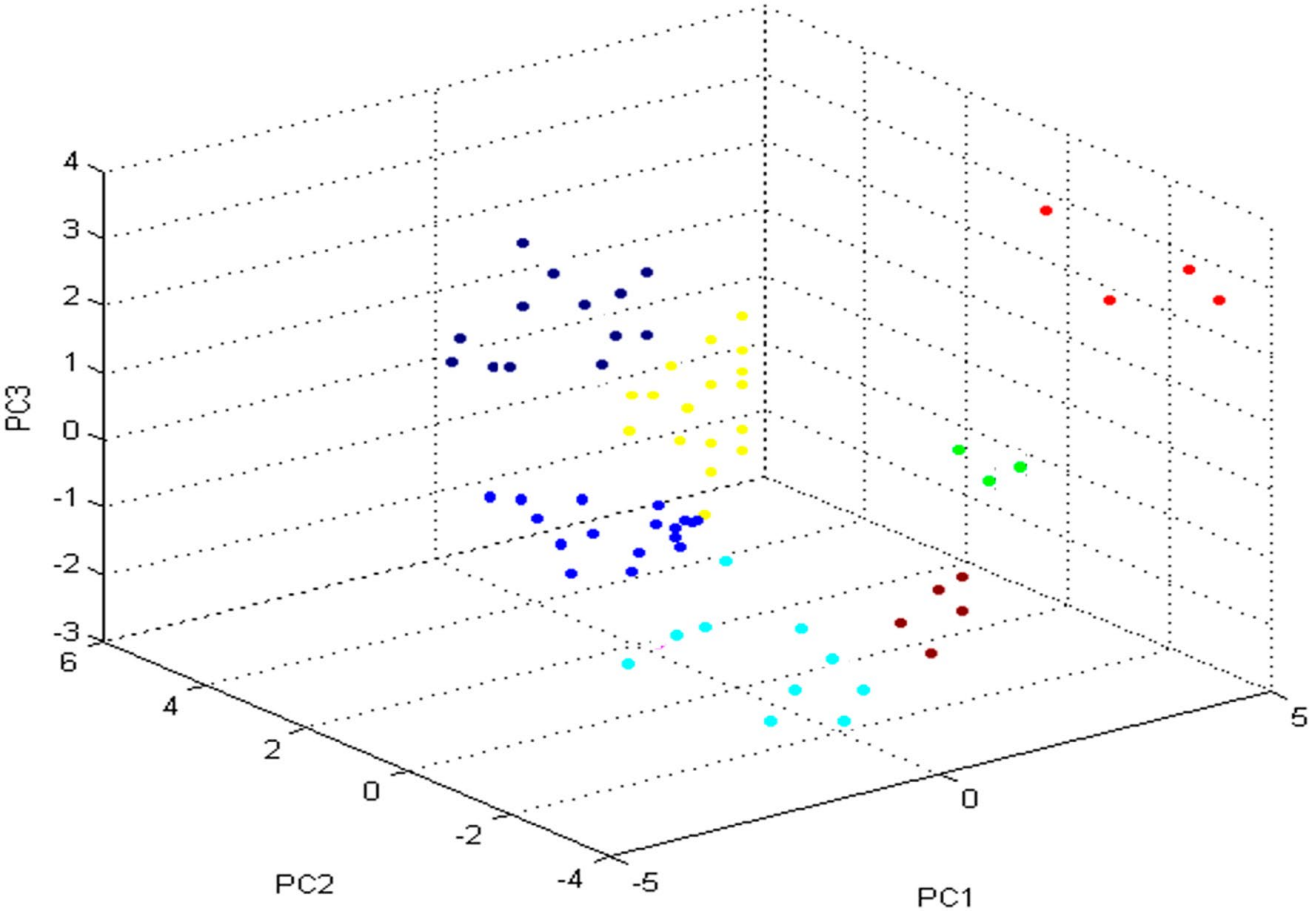
PCA dimensional image showing the grouping of 69 *Pseudomonas* spp.; **a** the alliance of *P. lundensis* (blue), *P. fragi* (yellow), *P. oryzihabitans* (blue black), *P. stutzeri* (aqua), *P*. *fluorescens* (maroon), *P. putida* (red), and *P. aeruginosa* (green) in the 1st three principal component models

As summarized in Table 4, we found that 10/18 (55.56%) *P. lundensis*, 7/16 (43.75%) *P. fragi*, 6/13 (46.15%) *P. oryzihabitans*, 4/10 (40%) *P. stutzeri,* 3/5 (60%) *P. fluorescens*, 1/4 (25%) *P. putida*, and 2/3 (66.67%) *P. aeruginosa* were properly recognized, with log values ranging from 2.3 to 3.0. Likewise, 8/18 (44.44%) *P. lundensis*, 9/16 (56.25%) *P. fragi*, 7/13 (53.85%) *P. oryzihabitans*, 5/10 (50%) *P. stutzeri,* 2/5 (40%) *P. fluorescens*, 3/4 (75%) *P. putida*, and 1/3 (33.33%) *P. aeruginosa* were properly identified, with log values ranging from 2.0 to 2.29. Nonetheless, one isolate of *P. stutzeri* was detected at the genus level with a log value ranging from 1.70 to 1.99. Zero strains were not detected. An additional calculation tool termed principal component analysis (PCA) was created in the present investigation in Microflex LT Compass IVD software to determine the similarities and differences between the spectral proteins of *Pseudomonas* spp. Several spectral proteins of various colours are presented in three‐dimensional (3d) images of PCA (Fig. 4). The 3 loading values originating from the calculation of PC1, PC2, and PC3 were used for the identification of each peak. In our examination, the wide-ranging peaks recorded in the Microflex LT Compass IVD software were evaluated by the PCA mathematical tool, which was able to separate the *Pseudomonas* strains by placing them in groups through dots of different colours (Fig. 4) as follows: *P. lundensis* (blue), *P. fragi* (yellow), *P. oryzihabitans* (blue black), *P. stutzeri* (aqua)*, P. fluorescens* (maroon), *P. putida* (red), and *P. aeruginosa* (green).

**Table 4:** Score values for 69 *Pseudomonas* spp. detected in chicken meat by PMFT

Species	Score value of identification							
	2.3–3		2–2.29		1.7–1.99		0–1.69	
	No.	%	No.	%	No.	%	No.	%
*P. lundensis*	10/18	55.56	8/18	44.44	0	0	0	0
*P. fragi*	7/16	43.75	9/16	56.25	0	0	0	0
*P. oryzihabitans*	6/13	46.15	7/13	53.85	0	0	0	0
*P. stutzeri*	4/10	40	5/10	50	1/10	10	0	0
*P. fluorescens*	3/5	60	2/5	40	0	0	0	0
*P. putida*	1/4	25	3/4	75	0	0	0	0
*P. aeruginosa*	2/3	66.67	1/3	33.33	0	0	0	0

A total of 69 different species of *Pseudomonas* were tested against 18 antimicrobial drugs utilized in the current investigation. According to the results labelled in Table 4, the majority of *Pseudomonas* species exhibited higher degrees of resistance against different classes of antibiotics. The highest degree of resistance was detected against nitrofuran (nitrofurantoin, 81.16%), followed by beta-lactam (ampicillin, 71%) and aztreonam, 55%), beta-lactam/beta-lactamase inhibitor (ampicillin/sulbactam, 71%), 2nd-generation cephalosporins (cefuroxime, 65.22%), 3rd-generation cephalosporins (ceftriaxone, 65.22%), ciprofloxacin (49.28%) and carbapenem (meropenem, 43.48%). In contrast, the most susceptible antimicrobial drugs were cefotaxime (100%) and ceftazidime (98.45%).

As shown in in Table 5, of 18 *P. lundensis* strains, 18 (100%), 17 (94.44%), 17 (94.44), 16 (88.89%, 16 (88.89%), 15 (83.33%) and 12 (66.67%) were resistant to ampicillin, amoxicillin/clavulanic acid, ampicillin/sulbactam, ciprofloxacin, nitrofurantoin, ceftriaxone, and cefuroxime, respectively. In contrast, all strains were sensitive to both cefotaxime and ceftazidime. In addition, out of 16 *P. fragi* strains*,* 16 (100%), 15 (93.75%), 15 (93.75%), 14 (87.5%), 13 (81.25%), 13 (81.25%), 12 (75%), and 12 (75%) were resistant to aztreonam, ceftriaxone, nitrofurantoin, ciprofloxacin, amoxicillin/clavulanic acid, cefuroxime, ampicillin, and cefoxitin, respectively. However, all strains of *P. fragi* showed higher degrees of susceptibility to only cefazolin, cefepime cefotaxime, and ceftazidime.

**Table 5:** Direct screening of the antimicrobial susceptibilities of certain Pseudomonas spp. by the VITEK AST GN83 cards

Antimicrobial agent	Percentage of antimicrobial resistance of *Pseudomonas* spp.														Total (n = 69)	
	*P. lundensis* (n = 18)		*P. fragi* (n = 16)		*P. oryzihabitans* (n = 13)		*P. stutzeri* (n = 10)		*P. fluorescens* (n = 5)		*P. putida* (n = 4)		*P. aeruginosa* (n = 3)			
	No.	%	No.	%	No.	%	No.	%	No.	%	No.	%	No.	%	No.	%
Amikacin	5	27.78	5	31.25	0	0.00	2	20.00	0	0.00	0	0.00	1	33.33	13	18.84
Amoxicillin/clavulanic acid	17	94.44	13	81.25	2	15.38	10	100.00	3	60.00	1	25.00	3	100.00	49	71.00
Ampicillin	18	100.0	12	75.00	1	7.69	10	100.00	3	60.00	2	50.00	3	100.00	49	71.00
Ampicillin/sulbactam	17	94.44	3	18.75	1	7.69	10	100.00	3	60.00	2	50.00	2	66.67	38	55.00
Aztreonam	2	11.11	16	100	0	0.00	1	10.00	0	0.00	0	0.00	1	33.33	20	28.99
Cefazolin	1	5.56	0	0.00	0	0.00	0	0.00	2	40.00	0	0.00	3	100.00	6	8.70
Cefepime	3	16.67	0	0.00	0	0.00	0	0.00	0	0.00	0	0.00	1	33.33	4	5.80
Cefotaxime	0	0.00	0	0.00	0	0.00	0	0.00	0	0.00	0	0.00	0	0.00	0	0.00
Cefoxitin	6	33.33	12	75.00	0	0.00	10	100.00	0	0.00	0	0.00	2	66.67	20	28.99
Ceftazidime	0	0.00	0	0.00	0	0.00	0	0.00	0	0.00	0	0.00	1	33.33	1	1.45
Ceftriaxone	15	83.33	15	93.75	3	23.08	10	100.00	0	0.00	0	0.00	2	66.67	45	65.22
Cefuroxime	12	66.67	13	81.25	5	38.46	10	100.00	2	40.00	1	25.00	2	66.67	45	65.22
Ciprofloxacin	16	88.89	14	87.50	0	0.00	3	30.00	0	0.00	0	0.00	1	33.33	34	49.28
Gentamicin	2	11.11	4	25.00	0	0.00	2	20.00	0	0.00	0	0.00	1	33.33	9	13.04
Meropenem	7	38.89	5	31.25	2	15.38	5	50.00	0	0.00	0	0.00	1	33.33	30	43.48
Nitrofurantoin	16	88.89	15	93.75	13	100.00	10	100.00	0	0.00	0	0.00	2	66.67	56	81.16
Piperacillin/Tazobactam	1	5.56	2	12.5	0	0.00	0	0.00	0	0.00	0	0.00	1	33.33	4	5.80
Trimethoprim/Sulfamethoxazole	2	11.11	4	25.00	0	0.00	0	0.00	0	0.00	0	0.00	1	33.33	7	10.14

Table 5 indicates that the majority of *P. oryzihabitans* isolates showed higher degrees of susceptibility for almost all antibiotics under study except nitrofurantoin, which exhibited a high resistance (100%), followed by cefuroxime (38.46%) and ceftriaxone (23.08%). Moreover, *P. stutzeri* strains exhibited a high resistance (100%) to amoxicillin/clavulanic acid, ampicillin, ampicillin/sulbactam, cefoxitin, ceftriaxone, cefuroxime, and nitrofurantoin. *P. fluorescens* strains also revealed a certain degree of resistance (60%) against amoxicillin/clavulanic acid, ampicillin, and ampicillin/sulbactam and 40% against cefazolin and cefuroxime. Fifty percent of *P. putida* strains were resistant ampicillin and ampicillin/sulbactam. Furthermore, 100% of *P. aeruginosa* strains were unaffected by amoxicillin/clavulanic acid, ampicillin, and cefazolin, and 66.67% were unaffected by ampicillin/sulbactam, cefoxitin, ceftriaxone, cefuroxime, and nitrofurantoin.

## Discussion

The contamination of chilled meat with various microbes is one of the major causes of cost-effective problems in meat production and has an impact on public health worldwide (Wickramasinghe et al. 2019). Despite the use of modern methods of meat preservation, microbial contamination remains a major threat. Psychrotrophic *Pseudomonas* spp. are considered the main bacteria that lead to putrefaction of ice-cold meat under aerobic conditions. The genus *Pseudomonas* is one of the most polluting microbes and is characterized by its great ability to withstand difficult environmental conditions, which leads to the prevention of the growth of other microorganisms. To diminish contamination of meat, proper detection and treatment of various *Pseudomonas* spp. are critical. Therefore, this study concentrated on an accurate method of identification and differentiation of *Pseudomonas* spp. and evaluated their degrees of resistance and susceptibility to various antibiotics commonly used for treatment.

Based on our results, the mean *Pseudomonas* counts (CFU/g) were 10.1 × 10^3^ ± 1.45 × 10^3^, 7.4 × 10^3^ ± 0.89 × 10^3^, 6.5 × 10^3^ ± 6.43 × 10^3^ and 4.3 × 10^3^ ± 7.56 × 10^3^ for the isolates recovered from the breast, thigh, burger, and nuggets, respectively. Similar findings were recorded by Hassan et al. (2020), who found that the mean *Pseudomonas* counts recovered from various chicken meat products (chilled breast, thigh, nuggets, and burger) varied from 3.51 × 10^3^ ± 0.76 × 10^3^ to 8.44 × 10^3^ ± 1.85 × 10^3^. Other parallel records detected by Morshdy et al. (2018) and Abd El-Aziz (2015) were 3.6 × 10^3^ and 2.6 × 10^4^, respectively. Although, the findings of the current study are similar to those of previous studies, the practice of comparing CFU/g of bacteria in meat samples across multiple studies is unusual because CFU is highly dependent on storage conditions, sampling preparation, and slaughter methods. Although the research performed by Hinton et al. (2007) indicated that psychrotrophic bacteria were not recovered from carcasses washed with chlorinated water, different species of *Pseudomonas* were the most predominant psychrotrophs recovered from all carcasses when stored in refrigerators for two weeks. Several investigations have revealed that the initial *Pseudomonas* count is directly associated with the period of storing meat in the refrigerator, and meat spoilage occurs when the number of *Pseudomonas* ranges from 10^7^ to 10^8^ (Hassan et al. 2020).

In the current investigation, we identified 69 *Pseudomonas* spp. using biochemical analysis confirmed by proteomics methods. The identified isolates were represented as *P. lundensis* (18), *P. fragi* (16), *P. oryzihabitans* (13), *P. stutzeri* (10), *P. fluorescens* (5), *P. putida* (4), and *P. aeruginosa* (3). Chicken meat burgers (25/69) and nuggets (25/69) were the most contaminated chicken meat products of *Pseudomonas* species, which might be a result of mismanagement, extreme usage, and unsuccessful hygienic practices throughout processing and packing. Parallel findings were obtained by Hassan et al. (2020), who identified 166 isolates of *Pseudomonas* species, with a high prevalence of *P. fluorescens* followed by *P. alcaligenes*, *P. stutzeri, P. proteolytica,* and *P. fragi,* while low incidence rates were recorded for *P. aeruginosa*, *P. stutzeri,* and *P. acidovorans*. In another study, Arnaut-Rollier et al. (1999) detected 3 species of *Pseudomonas* (*P. fragi*, *P. lundensis*, *P. fluorescens biovars*) from both fresh and refrigerated chicken skin.

In addition, 11 strains of *Pseudomonas* species recovered from cooked chicken burgers were identified by Franzetti and Scarpellini (2007) as 8 strains of *P. fragi*, followed by 2 *P. chicorii* and 1 *P. fluorescens*. In contrast, *P. aeruginosa* was not detected in 100 chicken meat specimens (Iroha et al. 2011). In another study conducted by Caldera et al. (2016), the deterioration of food was commonly associated with *P. aeruginosa*, *P. fragi*, *P. lundensis* and *P. fluorescens* (Caldera et al. 2016). Moreover, Bellés et al. (2017) and Wang et al. (2017) clarified that the capability of these microorganisms to live at low temperatures may lead to trouble throughout the storage of foodstuffs. The existence of *Pseudomonas* spp. in various food samples is of high importance because this type of bacteria has a bad impact on human health and is considered a sign of food quality (Yagoub 2009).

Because phenotypic‐based detection of various foodborne pathogens is difficult and takes a long time to be carried out, Microflex LT was meaningfully applied in our study for the initial detection and classification of numerous bacteria from chicken meat samples, as it is an easy, quick, specific, and inexpensive detection technique compared to other approaches (Singhal et al. 2015; van Belkum et al. 2017; Elbehiry et al. 2019). In recent times, Microflex LT has been discovered to be an imperative tool for the powerful recognition of microbial intimidations that may pollute both water and foodstuffs (Singhal et al. 2015; Elbehiry et al. 2019).

In the present investigation, the percentage of mass spectral identification of *Pseudomonas* strains was 100% for all 7 species of *Pseudomonas*. The interpreted results confirmed that all spectral profiles produced by Microflex LT IVD Compass Software were suitable to distinguish between *Pseudomonads* at the species level. The accurate identification observed in our study may be a result of the restructured database (Elbehiry et al. 2019). Comparable findings were noted by Böhme et al. (2011), who applied Microflex LT effectively in the exact identification of gram‐negative bacteria (e.g., *Pseudomonas* and *Enterobacter*) of different species recovered from seafood. Consequently, Microflex LT has been demonstrated to be an authoritative instrument for microbial identification. Höll et al. (2016) also identified several microorganisms isolated from packaged poultry meat using Microflex LT, and they found that *Pseudomonas* spp. is one of the most common bacteria found after 7 days of storage at 4 °C and 10 °C.

In addition, principal component analysis (PCA) generated by the Microflex LT device magnificently divided *P. lundensis, P. fragi, P. oryzihabitans, P. stutzeri, P. fluorescens, P. putida,* and *P. aeruginosa* strains into different groups. Han (2010) and Elbehiry et al. (2019) indicated that PCA is usually applied as a mathematical tool to extract and demonstrate the modification in the spectral profiles within the database.

The spread of antimicrobial resistance amongst the genus *Pseudomonas* was also examined in the current study. Of late, antimicrobial resistance represents one of the common public health problems, as multidrug-resistant bacteria related to animals may be virulent and transferred simply to human beings through food chains and comprehensively dispersed through animal wastes to the environment (Manyi-Loh et al. 2018). Antimicrobial resistance is problematic and multifaceted and occurs as a consequence of the unreasonable use of antibiotics under poor hygienic measures (Osman et al. 2019).

In the present investigation, the AST GN83 card was applied to display the resistance and susceptibility of 69 *Pseudomonas* species recovered from various chicken meat samples against several antibiotics frequently utilized for the treatment of gram‐negative pathogens. Based on our results, the majority of *Pseudomonas* isolates exhibited higher degrees of susceptibility to cefotaxime (100%), ceftazidime (98.55%), cefepime (94.2%), gentamycin (86.96%), and amikacin (82.16%). These findings were similar to those obtained by CLSI (2015). It was also observed that the majority of the *Pseudomonas* isolates were highly sensitive to meropenem (70%). Parallel results were achieved in previous studies carried out in Turkey by Shenoy et al. (2002) and Deniz Yilmaz et al. (2016) and in Kenya by Mwinyikombo (2018), who revealed that *Pseudomonas* isolates demonstrated higher degrees of susceptibility to both meropenem and imipenem. Nonetheless, other studies performed in India by Sivanmaliappan and Sevanan (2011) illustrated a higher degree of resistance to imipenem (66.6%), a finding that could be explained by the misuse of broad-spectrum antibiotics such as carbapenems.

The highest degree of resistance was detected against various classes of antibiotics, such as nitrofurantoin (81.16%), followed by beta-lactam [ampicillin (71%) and aztreonam (55%)], beta-lactam/beta-lactamase inhibitor [ampicillin/sulbactam (71%)], second-generation cephalosporins [cefuroxime (65.22%)], third-generation cephalosporins [ceftriaxone (65.22%)], ciprofloxacin (49.28%) and carbapenem [meropenem (43.48%)]. Similar results regarding resistance to nitrofurantoin were obtained by Sultana et al. (2014) and Agyare et al. (2018) who reported that *Pseudomonas* species from frozen foods of animal origin and poultry products, were resistant to nitrofurantoin. It can be seen from our study that the majority of *Pseudomonas* isolates were found to directly develop resistance against various types of antibiotics. According to our findings, *P. lundensis*, *P. fragi*, *P. oryzihabitans*, *P. stutzeri*, and *P. aeruginosa* may act as antibiotic resistance reservoirs.

There are many mechanisms through which pseudomonads gain multidrug resistance, including reduced outer membrane permeability (De Oliveira et al. 2013; Lavilla Lerma et al. 2014), beta-lactamase production, and multidrug efflux pumps with a broad substrate spectrum (Henwood et al. 2001; Lavilla Lerma et al. 2014). It has been suggested that the use of antimicrobials that can enhance gene transfer by enhancing the SOS system (Lima et al. 2020), as well as the presence of pathogens as potential reservoirs of resistance factors, may all contribute to an increase in antibiotic resistance in pseudomonas. Pathogenic bacteria like pseudomonas can grow in a wide variety of habitats, each of which has important variables that contribute to the evolution of their resistance (Pachori et al. 2019). Because many resistance genes are carried on plasmids or integrons, the spreading of multiple drug-resistant pseudomonads from various sources to both people and the environment strongly suggests horizontal gene transfer as the primary pathway for dissemination of resistance determinants (Von Wintersdorff et al. 2016).

Based on the results of our study, we demonstrate the high incidence rate of *P. lundensis*, *P. fragi*, and *P. oryzihabitans* among different *Pseudomonas* species found in chicken meat samples. Microflex LT for the detection of *Pseudomonas* species proved to be a reliable, affordable, and easy-to-apply method during this investigation and PCA generated by Microflex LT enabled discrimination between different species of *Pseudomonas*. In future studies, it will be necessary to determine if this technique can be useful in recognizing and correcting discrepancies in *Pseudomonas* spp. in food samples. We also found that the various species of *Pseudomonas* are multidrug resistant. In this way, it is conceivable that resistance will evolve over time, which is why the number of antimicrobials is decreasing. Due to the potential threat posed by *Pseudomonas*, the transmission of resistance may negatively affect individuals.

## Data Availability

The data that support the findings of this study are available on request from the corresponding author.

## Abbreviations

PCA: Principal component analysis
IVD: In vitro diagnostic device
